# Supplementary figures and images for: Deficiency of Crif1 in hair follicle stem cells retards hair growth cycle in adult mice
Source: PLoS One. 2020 Apr 24;15(4):e0232206. doi: 10.1371/journal.pone.0232206 (PMC7182249; doi:10.1371/journal.pone.0232206)

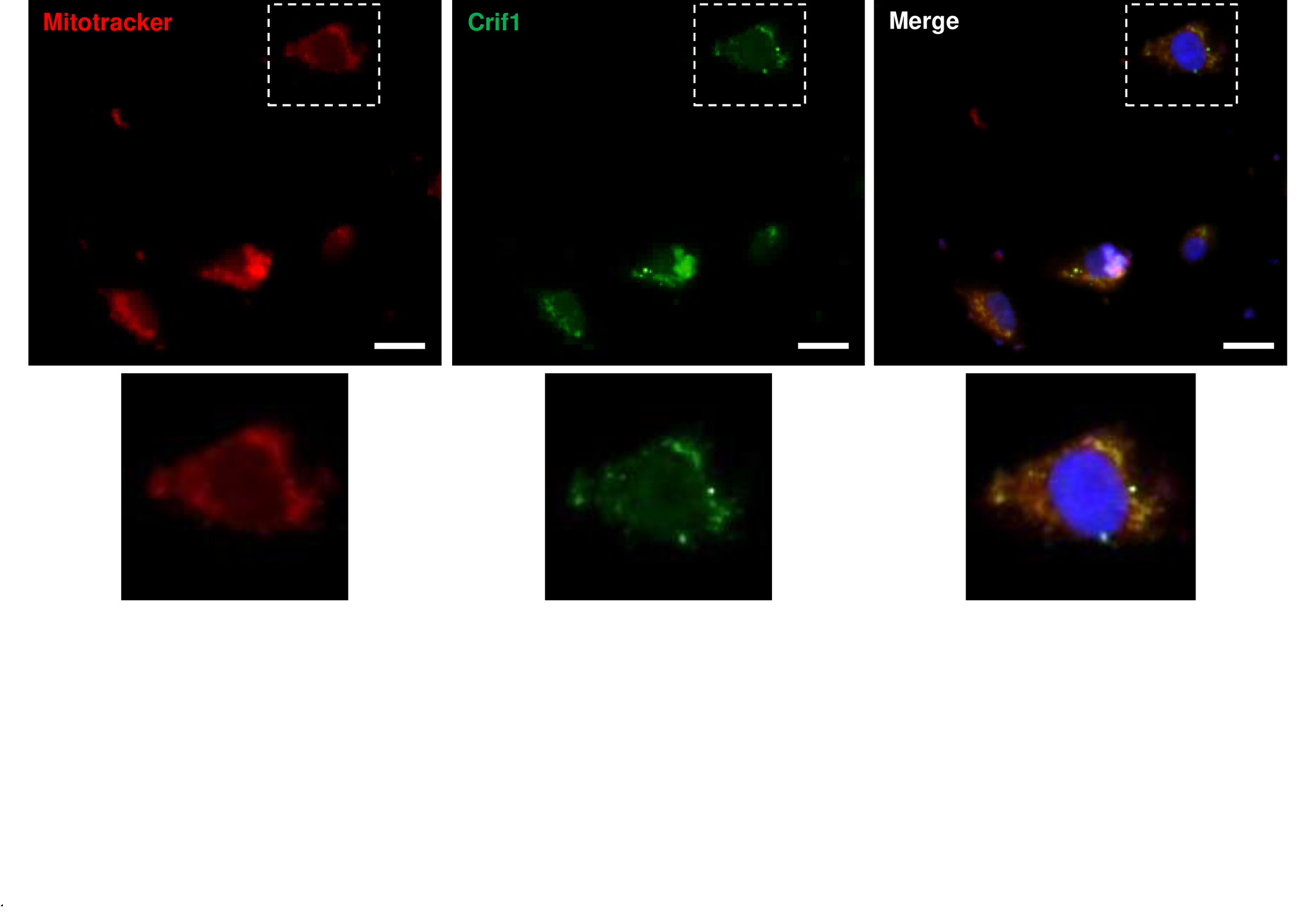

Supplement: S1 Fig — Cultured outer root sheath (ORS) cells were co-stained with mitotracker (red) and Crif1 (green). Nucleus was counterstained with 4,6-diamidino-2-phenylindole (DAPI, blue). Scale bar, 20 μm. (TIF) [file pone.0232206.s001.tif]

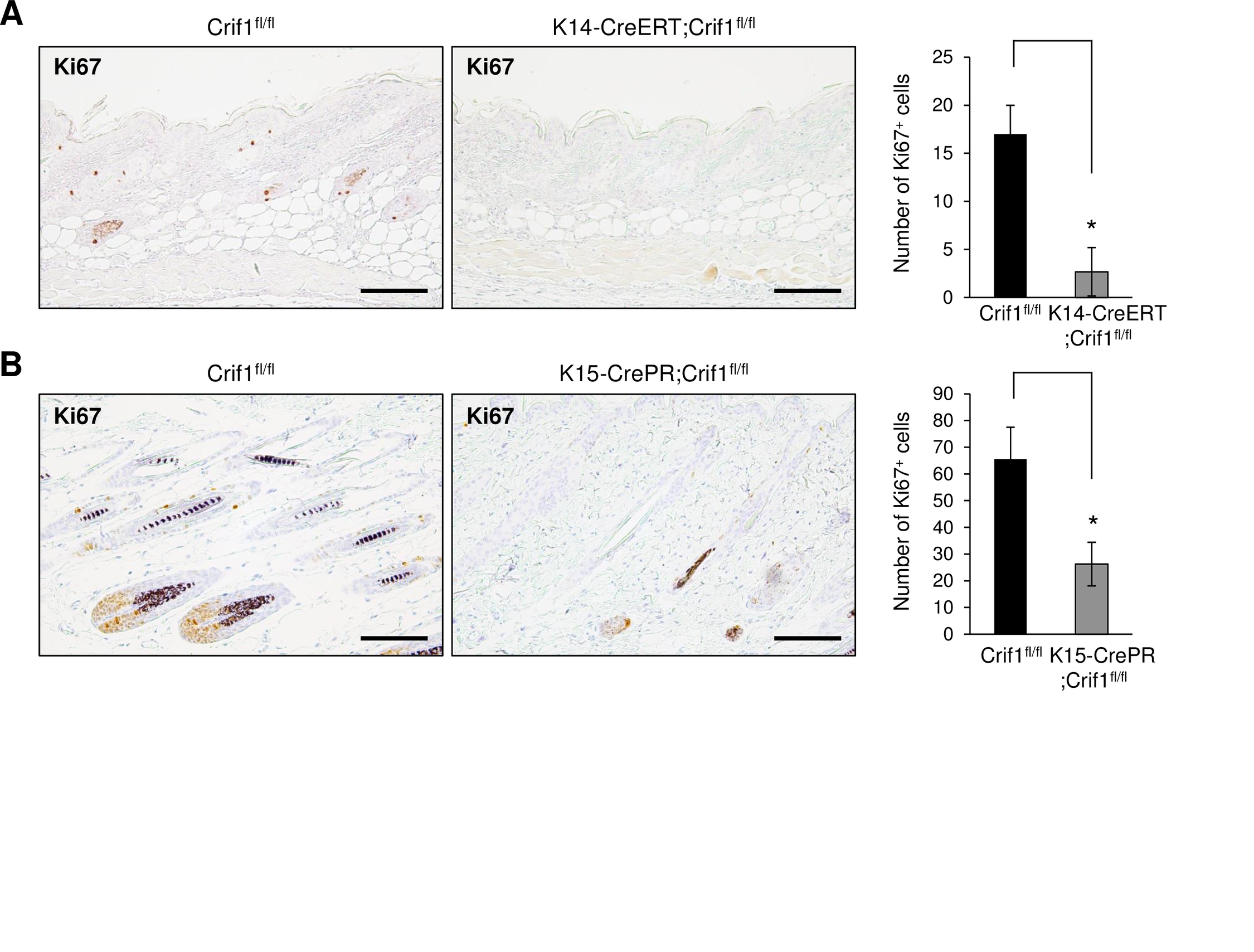

Supplement: S2 Fig — (A) Crif1fl/fl (WT) mice and K14-CreERT;Crif1fl/fl (Crif1 K14icKO) mice were shaved at P21 and topically applied with 4-hydroxy tamoxifen (1 mg/mice) for 5 days. Skin sections were obtained at P35 and stained using Ki67 antibody. (B) Crif1fl/fl mice and K15-CrePR;Crif1fl/fl (Crif1 K15icKO) mice were shaved at P21 and topically treated with RU486 (1 mg/mice) for 5 days. Skin sections were obtained at P44 and stained using Ki67 antibody. Scale bar, 100 μm. (TIF) [file pone.0232206.s002.tif]

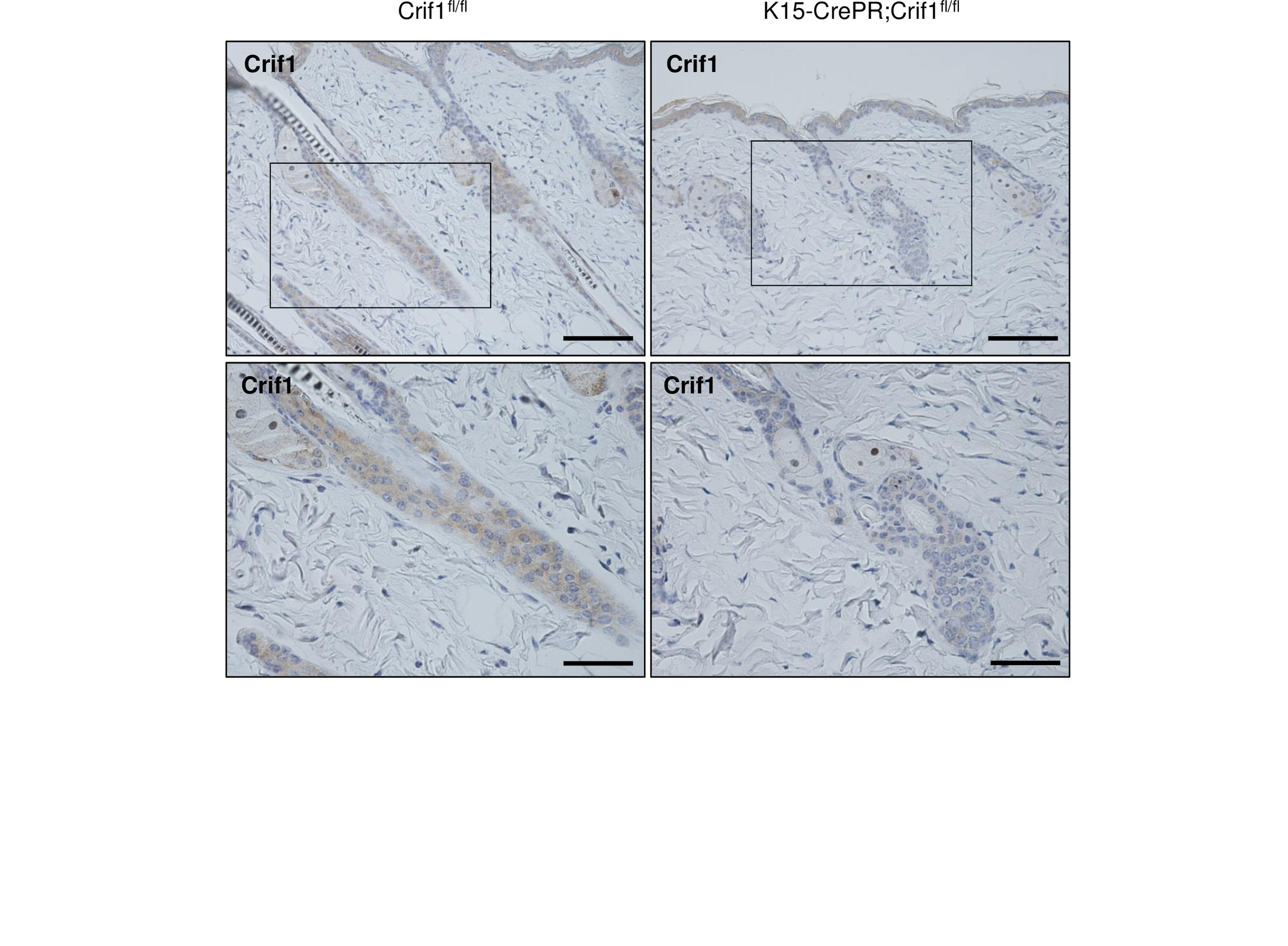

Supplement: S3 Fig — Skin sections were obtained at P44 and stained using Crif1 antibody. Crif1 immunoreactivity was observed in epidermis of Crif1 K15icKO mice. In contrast, Crif1 immunoreactivity was very weak in hair bulge of Crif1 K15icKO mice. Scale bar, 100 μm (upper), 50 μm (lower). (TIF) [file pone.0232206.s003.tif]

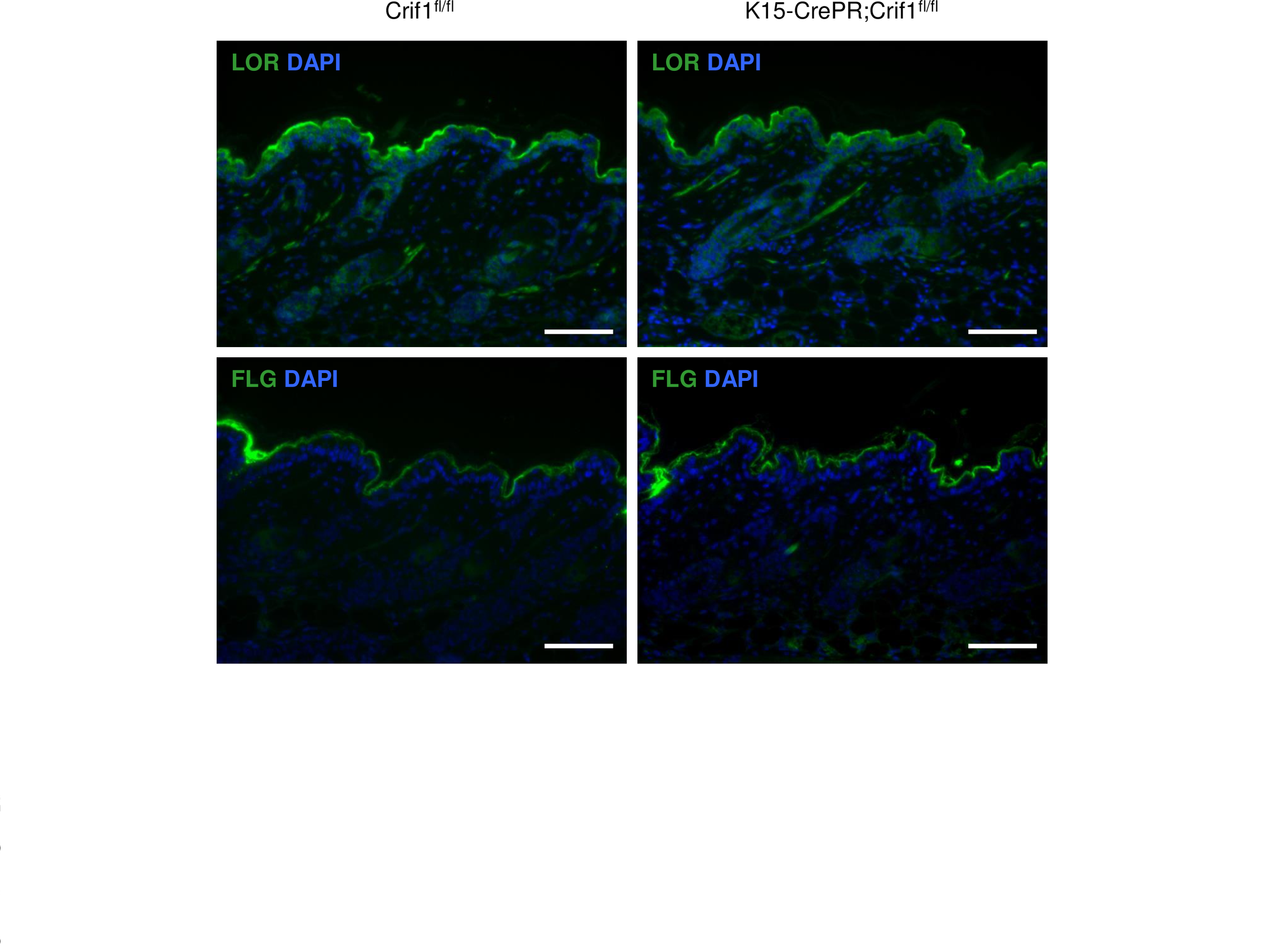

Supplement: S4 Fig — Crif1fl/fl (WT) mice and K15-CrePR;Crif1fl/fl (Crif1 K15icKO) mice were shaved at P21 and topically treated with RU486 (1 mg/mice) for 5 days. Skin sections were obtained at P44 and stained using loricrin (LOR) and filaggrin (FLG) antibodies. There was no difference in differentiation marker expression between WT and Crif1 K15icKO mice. Scale bar, 100 μm. (TIF) [file pone.0232206.s004.tif]

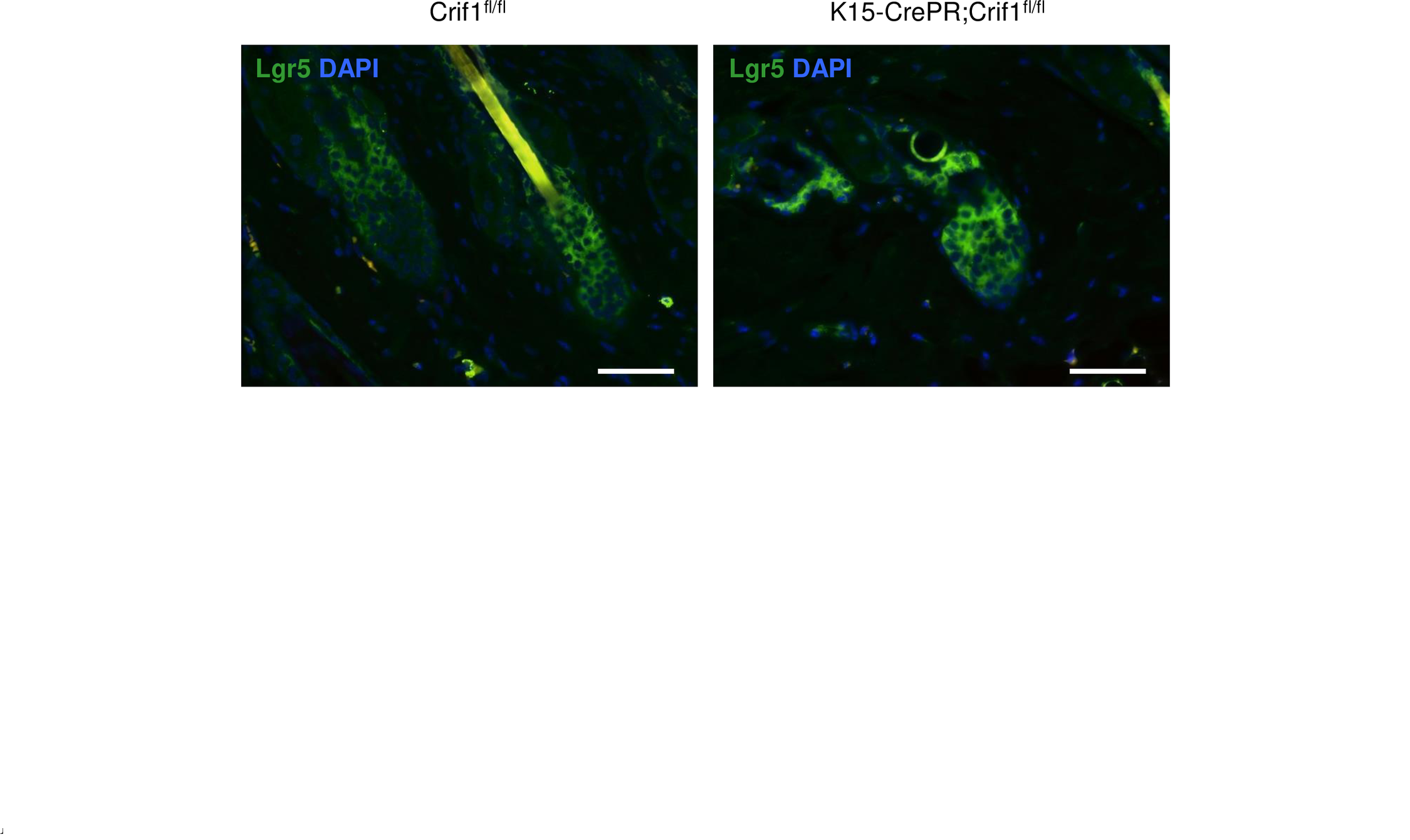

Supplement: S5 Fig — Skin sections were obtained at P44 and stained using Lgr5 antibody. Lgr5 was detected in both the WT and Crif1 K15icKO mice. Scale bar, 50 μm. (TIF) [file pone.0232206.s005.tif]

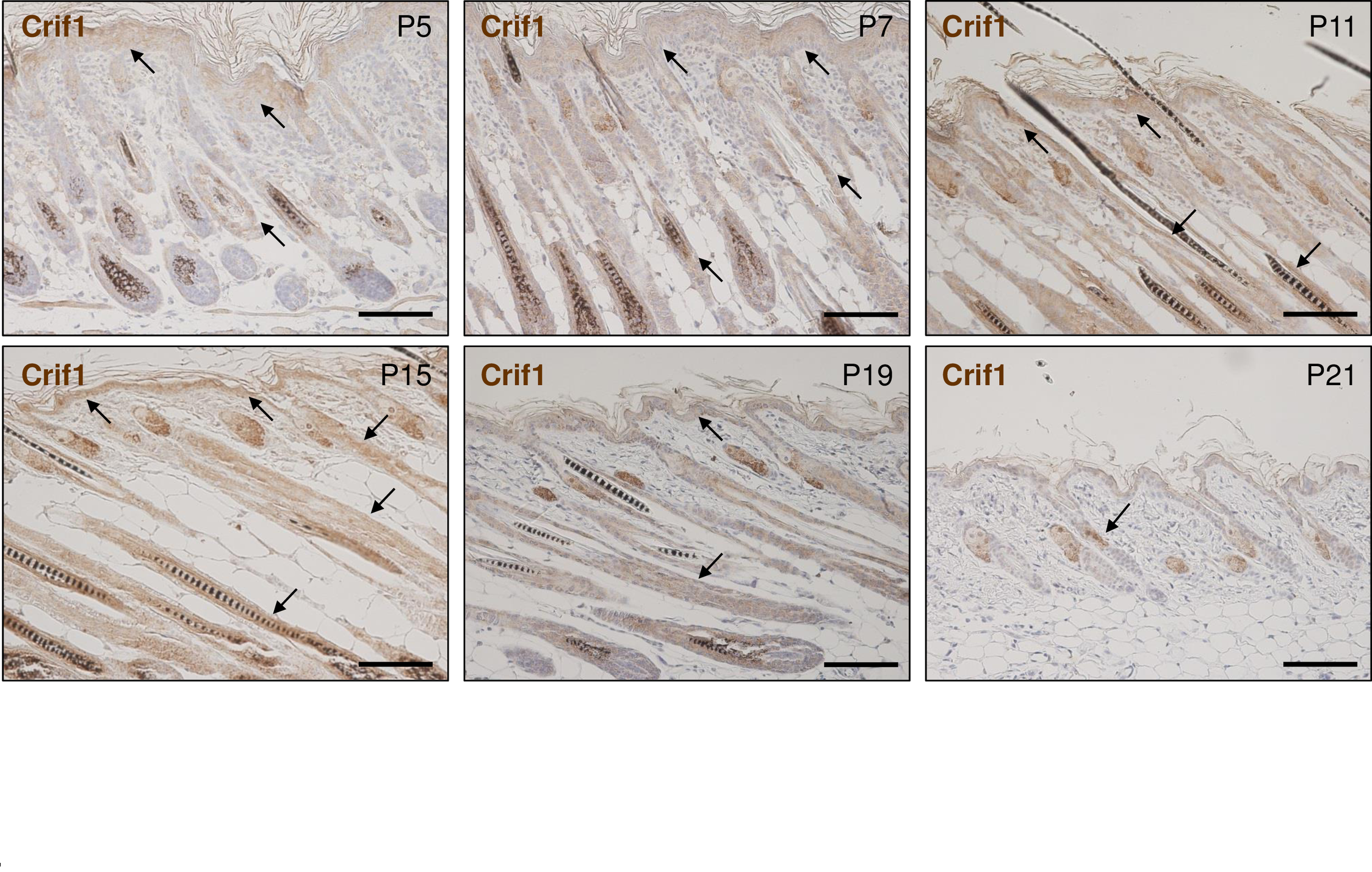

Supplement: S6 Fig — Scale bar, 200 μm. (TIF) [file pone.0232206.s006.tif]
