# Supplementary material for: Deficiency of Crif1 in hair follicle stem cells retards hair growth cycle in adult mice
Source: PLoS One. 2020 Apr 24;15(4):e0232206. doi: 10.1371/journal.pone.0232206 (PMC7182249; doi:10.1371/journal.pone.0232206)
Supplement: Attachment — Submitted filename: Response to Reviewer_rev2.docx [file pone.0232206.s008.docx]

**Response to Reviewer**

**Reviewer #1: The authors have adequately addressed most comments raised in the previous round of review however there is still a point that I think it should be taken in consideration.
Since the Crif1 deletion does not lead to a loss of HF stem cells, while some defects of HF differentiation have been observed (AE15 staining), I think that the interpretation of the Crif1 inducible KO phenotype is linked to lower efficiency to produce differentiated HF progenies by the HF stem cells.
In this direction, only the AE15 staining has been performed. I suggest to add the staining for another HF differentiation marker and quantify the result from the two markers.**
As you suggested, we performed additional immunohistochemical staining using AE13 antibody obtained from Abcam (ab16113) to detect hair cortex. AE13 immunoreactivity in Crif1 K15icKO mice was markedly weaker than that of WT mice. This result supported that Crif1 deletion induces defects of hair follicle differentiation. We added new data in revised Fig 2D. We also added the descriptions for Fig 2D in page 10 / line 4, 6 and page 10 / line 23 of revised manuscript.

**Minor point:
The sentence in the text about K5 is meaningless: ”However, it was likely that K5 immunoreactivity in Crif1 K15icKO mice was slightly weaker than that of WT mice ”.**
We agreed with your point, and deleted the sentence in revised manuscript.

**Reviewer #2: The authors have addressed almost all the concerns I have raised in the first review. They performed experiments to prove that the quiescent cell populations were not affected by Crif1 deletion which further reinforces that Crif1 is dispensable for HFSC. Proliferation assay using Ki67 antibody further validates that the Crif1 deletion impedes the** **proliferation, thus contributing to hair cycle retardation likely by affecting proliferative stem cell population. The manuscript has been improved well and can be published in PLoS One upon minor revision on following;**

**Fig 4A – Add label for DAPI**

We added label for DAPI in Fig 4A.

**Fig S2 – Very difficult to infer Ki67 expression from these images. Quantification must be done. Add label for Ki67 in each image.**

We quantified Ki67 positive cells and added graphs. We also added label for Ki67 in each image of Fig S2.

**Fig S3 – Area of magnification belongs to lower panel should be indicated in the upper panel.**

We marked area of magnification in the upper panel of Fig S3.

**Fig S4 – Add label for DAPI in each image.**
We added label for DAPI in Fig S4.

**Fig S5 – Add label for Lrig1 and DAPI in each image.**
We added label for Lrig1 and DAPI in Fig S5.

**Fig S6 – Add label for Crif1 in each image and indicate its site of expression by arrow.**

We added label for Crif1 and indicated expression of Crif1 by arrow in Fig S6.

**Individual data points in all graphs – I meant to include data points (dots) for each replicate, not the “cumulative value”. For example, if n=3 is carried out in an experiment, the distribution of each value represented as data points(=dots) should be overlaid on the error bar in a bar graph.**

As your comments, we changed all graphs including data points. (Fig 1A, Fig1D, Fig 2C, Fig 3C, Fig 4A and Fig 4B)
